# Special supplement issue on quality assurance and enrichment of biological and biomedical ontologies and terminologies

**DOI:** 10.1186/s12911-024-02654-5

**Published:** 2024-08-30

**Authors:** Licong Cui, Ankur Agrawal

**Affiliations:** 1 McWilliams School of Biomedical Informatics, The University of Texas Health Science Center at Houston, Houston, TX USA; 2 Department of Computer Science, St. Edward’s University, Austin, TX USA

**Keywords:** Ontology, Quality assurance, Auditing, Enrichment, Application

## Abstract

Ontologies and terminologies serve as the backbone of knowledge representation in biomedical domains, facilitating data integration, interoperability, and semantic understanding across diverse applications. However, the quality assurance and enrichment of these resources remain an ongoing challenge due to the dynamic nature of biomedical knowledge. In this editorial, we provide an introductory summary of seven articles included in this special supplement issue for quality assurance and enrichment of biological and biomedical ontologies and terminologies. These articles span a spectrum of topics, such as development of automated quality assessment frameworks for Resource Description Framework (RDF) resources, identification of missing concepts in SNOMED CT through logical definitions, and developing a COVID interface terminology to enable automatic annotations of COVID-19 related Electronic Health Records (EHRs). Collectively, these contributions underscore the ongoing efforts to improve the accuracy, consistency, and interoperability of biomedical ontologies and terminologies, thus advancing their pivotal role in healthcare and biomedical research.

## Background

Ontologies and terminologies play a critical role in the systematic representation of knowledge in biomedicine. They not only serve as a part of the metadata standards for describing data in the FAIR Data Principles (Findable, Accessible, Interoperable, Reusable) [1], but also play a vital role in downstream applications as a declarative knowledge source [2, 3]. For example, SNOMED CT [4], the most comprehensive and precise clinical health terminology in the world, facilitates the clear exchange of health information in Electronic Health Records (EHRs), leading to higher quality, consistency and safety in healthcare delivery [5, 6].

As biomedical ontologies and terminologies grow in size and complexity, quality assurance and enrichment become increasingly essential to ensure their accuracy, consistency, interoperability, and usability across expanding domains and applications in healthcare and biomedical research. There have been several literature review articles that focused on the methodologies for auditing and quality assurance of biomedical terminologies and ontologies. In 2009, Zhu et al. [7] conducted an extensive review of early auditing methods for biomedical terminologies. Amith et al. [8] subsequently surveyed newer quality assurance approaches for biomedical ontologies published between 2009 and 2017. Zheng et al. [9] conducted an in-depth review of auditing methods for the Unified Medical Language System (UMLS), including ontology enrichment and alignment techniques. Additionally, Geller et al. have organized two special issues [10, 11] in 2009 and 2018, featuring advanced methods for auditing and quality assurance of biomedical terminologies. In 2020, we organized a special issue dedicated to quality assurance and enrichment of biological and biomedical ontologies and terminologies [12].

In this new special supplement issue, we solicited and selected articles capturing more recent developments related to quality assurance and enrichment of biological and biomedical ontologies and terminologies. We invited submissions by distributing calls for papers to major listservs. Following a rigorous single-blind review process, seven articles [13–19] were accepted for publication, each reviewed by two or more reviewers with relevant expertise.

## Summary of articles in this special supplement issue

The paper “Mining of EHR for interface terminology concepts for annotating EHRs of COVID patients” [13] presents the development of a COVID Interface Terminology (CIT) aimed at enhancing the automatic annotation of Electronic Health Records (EHRs) related to COVID-19, addressing gaps in existing terminologies and improving the quality of annotations. The paper highlights the challenges in leveraging unstructured EHR data due to insufficient annotations, which impedes the automatic extraction of useful information from unstructured text. To overcome these challenges, CIT was developed by integrating existing COVID-related ontologies and mining additional, more granular concepts from clinical notes using techniques such as anchoring and concatenation. The study demonstrates that CIT provides significantly better annotation coverage compared to existing ontologies like SNOMED CT and Coronavirus Infectious Disease Ontology (CIDO), with about 20% more coverage than SNOMED CT and 50% more than CIDO. This improved annotation accuracy is expected to facilitate more effective information extraction from EHRs, benefiting both research and clinical decision-making. Furthermore, the mined concepts within CIT could serve as valuable training data for machine learning models, potentially leading to even greater coverage and utility in the future.

The paper “Logical definition-based identification of potential missing concepts in SNOMED CT” [14] introduces a systematic approach to identifying potential missing concepts in SNOMED CT. The approach intersects logical definitions from unrelated, fully defined concepts in non-lattice subgraphs, generating new logical definitions that may represent missing concepts. A fine-tuned PEGASUS text summarization model is then used to predict fully specified names for these potential missing concepts. The approach not only provides logical definitions for the missing concepts but also predicts their fully specified names, aiming to enhance the completeness and accuracy of the ontology. The approach was applied to the March 2021 US Edition of SNOMED CT resulting in 30,313 unique logical definitions, from which 23,031 potential missing concepts were identified, with 10.04% automatically validated using external resources like UMLS, PubMed, and a newer SNOMED CT version. The findings suggest the approach is promising but requires further enhancement in naming concepts based on logical definitions.

The paper “Big knowledge visualization of the COVID-19 CIDO ontology evolution” [15] focuses on addressing the challenges of visualizing and understanding the evolution of the Coronavirus Infectious Disease Ontology (CIDO). CIDO, being the largest and most rapidly growing COVID-19 ontology, poses difficulties for researchers who need to stay updated on its frequent changes and how these updates are relevant to their specific research needs. The paper introduces a new visualization framework called Diff Weighted Aggregate Taxonomy (DWAT), which builds on the Weighted Aggregate Taxonomy (WAT) to provide a “big picture” view of the differences between two releases of the CIDO ontology, helping researchers quickly grasp the evolution of the ontology. Additionally, the DWAT framework supports a layered approach, allowing users to begin with a broad overview and progressively delve into more detailed, specific topics of interest. The paper demonstrates the use of DWAT to analyze the evolution of CIDO between 2020 and 2022, highlighting the ontology’s growth and changes over time, enabling users to quickly grasp the most significant updates and explore finer details when necessary.

The paper “Automated approach for quality assessment of RDF resources” [16] presents an automated quality assessment framework for Resource Description Framework (RDF) resources, particularly focusing on foundational metrics across three categories: Resolvability, Parsability, and Consistency. By curating 61 automatable metrics and selecting six foundational ones, the authors developed an open-source tool to assess RDF resources, identifying issues such as non-resolvable Unique Resource Identifiers (URIs) and undefined URIs. The tool was applied to eight widely-used RDF resources in healthcare, including HL7 FHIR and CDISC CDASH. The results reveal varying levels of unresolved URIs and the absence of errors in parsability and consistency metrics. The findings suggest that the automated quality assessment tool is effective in identifying RDF resource quality issues and can be expanded to include additional quality metrics.

In the article “An ontology-based approach for harmonization and cross-cohort query of Alzheimer’s disease data resources” [17], the authors developed an ontology-based approach to harmonize and enable cross-cohort queries of Alzheimer’s disease (AD) data resources from the National Alzheimer’s Coordinating Center (NACC) and the Alzheimer’s Disease Neuroimaging Initiative (ADNI), two major Alzheimer’s Disease research resources in the United States. By mapping data elements between NACC and ADNI, harmonizing inconsistent permissible values, and creating the Alzheimer’s Disease Data Element Ontology (ADEO), the authors identified 172 mappings and constructed common concepts. A prototype cross-cohort query system was developed, comprising a web-based interface, an advanced query engine, and a MongoDB database backend, to facilitate searching patient cohorts across NACC and ADNI. The work not only aimed to enhance data harmonization and interoperability between these two major AD research resources, but also laid the groundwork for potential application in other domains for querying patient cohorts from diverse data sources.

The paper “DEVO: an ontology to assist with dermoscopic feature standardization” [18] discusses the development of an ontology called Dermoscopy Elements of Visuals Ontology (DEVO), which aims to standardize the terminology used in dermoscopic analysis for diagnosing skin diseases. The paper emphasizes the importance of dermoscopy, a non-invasive technique used to examine pigmented skin lesions and improve diagnosis accuracy. However, the rapid evolution and proliferation of dermoscopic vocabulary without standardized control have led to inconsistencies and redundancies within the field. To address these issues, the authors developed DEVO, a domain-specific ontology that formalizes the definitions of dermoscopic metaphorical terms by decomposing them into their visual elements. The ontology is built in two phases: the first phase involves creating a foundational ontology (Elements of Visuals Ontology or EVO) that covers basic aspects of visualization, such as shapes, colors and patterns. The second phase involves creating the domain ontology (DEVO) that harnesses EVO to formalize the definitions of dermoscopic metaphorical terms. DEVO includes 1,047 classes, 47 object properties, and 16 data properties, and it was found to demonstrate a higher semiotic score compared to similar ontologies. The paper highlights the potential applications of DEVO in educating trainees, supporting dermatologists in making diagnosis, and facilitating the standardized exchange of knowledge in the dermoscopy domain.

The paper “Strategy maintenance in smart healthcare systems” [19] introduces TAnom-HS, an approach designed to manage anomalies within healthcare strategies, focusing on improving the accuracy and efficiency of knowledge representation and inference processes in smart healthcare systems. The approach consists of two main steps: extracting relationships between statements and resolving anomalies. TAnom-HS aims to enhance the quality of decision-making in medical scenarios by addressing issues such as conflicts, redundancies, cycles, and inaccessible statements. By developing and testing a prototype on cases from the BioPortal repository, the authors demonstrated the effectiveness of TAnom-HS in detecting and addressing anomalies, thus facilitating more reliable and efficient decision-making in healthcare. The authors also identified gaps in current tools and methods for managing healthcare strategies and suggested future research directions to enhance strategy verification tools, improve anomaly resolution techniques, and explore machine learning applications to optimize decision-making processes in healthcare systems.

## Conclusions

The articles selected in this special supplement issue collectively advance the field of biomedical ontologies and terminologies by addressing critical challenges in quality assurance, enrichment, and application. As biomedical data continues to grow in complexity and volume, the importance of accurate, consistent, and interoperable terminologies becomes increasingly vital. The innovative methodologies and tools presented in these papers, ranging from mining Electronic Health Records for enhanced clinical terminologies to developing automated quality assessment frameworks for RDF resources, demonstrate significant strides in enhancing the robustness and usability of biomedical ontologies. Looking ahead, these efforts set a strong foundation for ongoing research and development in quality assurance and enrichment of biomedical ontologies. They are essential in ensuring that biomedical ontologies and terminologies can keep pace with the evolving needs of healthcare and research. We anticipate that the ideas and innovations presented in these studies will inspire future research and practical applications, further advancing the field and enhancing the quality of healthcare delivery and biomedical discovery.

## Data Availability

Not applicable.

## Abbreviations

AD: Alzheimer’s disease
ADEO: Alzheimer’s Disease Data Element Ontology
ADNI: Alzheimer’s Disease Neuroimaging Initiative
CIDO: Coronavirus Infectious Disease Ontology
CIT: COVID Interface Terminology
DEVO: Dermoscopy Elements of Visuals Ontology
DWAT: Diff Weighted Aggregate Taxonomy
EHR: Electronic Health Record
EVO: Elements of Visuals Ontology
FAIR: Findable, Accessible, Interoperable, Reusable
NACC: National Alzheimer’s Coordinating Center
RDF: Resource Description Framework
UMLS: Unified Medical Language System
URI: Unique Resource Identifier
WAT: Weighted Aggregate Taxonomy
